# Performance of Nursing Activities Score in intensive care units of a teaching hospital in a low-middle-income country: An observational study

**DOI:** 10.1371/journal.pgph.0006169

**Published:** 2026-08-24

**Authors:** Raj Kumar Mehta, Hafizah Che Hassan, Bishnu Bista, Mamata Sharma Neupane

**Affiliations:** 1 Department of Nursing, Lincoln University College, Petaling Jaya, Selangor, Malaysia; 2 Department of Nursing, Chitwan Medical College Teaching Hospital, Chitwan, Nepal; University of Colorado Anschutz Medical Campus: University of Colorado - Anschutz Medical Campus, UNITED STATES OF AMERICA

## Abstract

Nursing workload in intensive care units (ICUs) is a key determinant of patient outcomes, quality of care, and efficient resource utilization. The Nursing Activities Score (NAS) is a validated instrument for quantifying nursing workload; however, evidence regarding its use in low- and middle-income countries remains limited. This observational study assessed nursing workload using the NAS and evaluated its ability to predict patient outcomes among 501 adult patients admitted to the ICU of a teaching hospital between 7 March 2022 and 27 February 2023. NAS was recorded for each patient, and outcomes were classified as survivors and non-survivors. The predictive performance of NAS was evaluated using receiver operating characteristic (ROC) curve analysis, model calibration was assessed with the Hosmer–Lemeshow goodness-of-fit test, and logistic regression was used to examine the association between NAS and mortality. Differences in NAS scores between survivors and non-survivors were compared using the Mann–Whitney U test. The median NAS was 83.40 (IQR: 68.30–101.10; range: 39.2–134.4), indicating a high nursing workload. NAS demonstrated good discriminative ability for predicting patient outcomes (AUROC = 0.838, p < 0.001), with an optimal cut-off value of 90.40, yielding 73.5% sensitivity and 73.1% specificity. The model showed good calibration (Hosmer–Lemeshow p = 0.422). Higher NAS scores were significantly associated with mortality (Exp(B) = 0.937, p < 0.001), and non-survivors had significantly higher NAS scores than survivors (110.70 vs. 76.20, p < 0.001). These findings demonstrate that the NAS is a reliable tool for measuring ICU nursing workload and predicting patient outcomes, supporting its use to optimize nurse staffing, workload allocation, and critical care resource planning in resource-limited settings.

## 1. Introduction

Nursing workload in intensive care units (ICUs) is a crucial factor influencing patient safety, quality of care, and nurse well-being. The complexity of care in ICUs demands continuous monitoring, invasive procedures, medication administration, and emergency interventions, making nurse staffing and workload key determinants of patient outcomes [1,2]. Nurse staffing shortages and excessive workload have been associated with higher mortality rates, increased nosocomial infections, longer ICU stays, and increased burnout among nurses [3,4]. A study by Kartik et al. (2017) reported that nursing staff allocation is a major problem in many ICUs and hospitals in India [5]. Patil et al. (2023) further emphasized that a higher mortality rate found in an ICU of a public teaching hospital in India and attributed this finding to the low nurse-to-patient ratio due to nurse shortage, budgetary constraints, and higher nursing workload [6]. However, in low-middle-income countries (LMICs) like Nepal, meeting the recommended 1:1 nurse-to-patient ratio remains a significant challenge [7]. Consequently, nurses often manage multiple critically ill patients simultaneously, leading to increased workload, compromised patient safety, and suboptimal adherence to clinical protocols [8]. Adequate nurse staffing is essential for maintaining high-quality patient care in ICUs. Studies indicate that higher nurse-patient ratios lead to better patient outcomes, while staff shortages increase the risk of mortality and medical errors [1,9]. A systematic review by Kane et al. (2007) confirmed that an increase in registered nurse staffing is associated with lower hospital mortality, reduced infections, and improved recovery rates [4]. Similarly, Numata et al. (2006) found that ICUs with lower nurse-to-patient ratios had higher mortality rates, underscoring the importance of appropriate staffing in critical care settings [10]. In many LMICs, the ICU nurse-to-patient ratio often exceeds the recommended standards, sometimes reaching 1:3 or more due to staff shortages and limited resources [7]. This excessive workload not only increases the risk of burnout among nurses but also jeopardizes patient safety and care quality [8]. Park J et al. (2022) reported that excessive nursing workload leads to cognitive overload, which can impair clinical decision-making and increase the likelihood of medical errors [2]. In such settings, tools for objectively measuring nursing workload are essential for workforce management and quality improvement initiatives. The Nursing Activities Score (NAS) is a validated tool used to measure nursing workload in ICUs. Developed by Miranda et al. (2003), the NAS is widely used in high-income countries to quantify the time and effort required for nursing care in critically ill patients [11]. The NAS assigns numerical values to different nursing activities, providing an objective measure of workload based on patient needs. Studies have shown that higher NAS scores correlate with increased nursing workload and patient severity [12]. However, despite its reliability, NAS has not been widely studied in LMICs, including Nepal, where ICU conditions and staffing challenges differ significantly from those in high-income countries. A study by Oliveira AC et al. (2016) found that NAS is a strong predictor of nursing workload, with higher NAS scores associated with increased nursing interventions and patient complexity [12]. Additionally, Carayon P et al. (2014) emphasized that measuring nursing workload is essential for maintaining patient safety, as excessive workload can lead to nurse fatigue, reduced vigilance, and an increased risk of errors. While NAS has been validated in various ICU settings, its application in resource-limited environments remains unexplored, highlighting a crucial knowledge gap in workload assessment in Nepalese ICUs [7]. A growing body of research links higher nursing workload to increased patient mortality. West et al. (2014) demonstrated that higher ICU nurse staffing levels were associated with lower mortality rates, suggesting that adequate nurse staffing is a protective factor against adverse outcomes [9]. Numata et al. (2006) further confirmed that ICU mortality rates are significantly influenced by nurse workload, particularly in high-acuity patients requiring intensive interventions [10]. Additionally, nursing workload has been associated with an increased risk of adverse events, including nosocomial infections, pressure ulcers, medication errors, and delayed interventions [4]. In ICUs with high nurse workload, infection rates are significantly higher, as nurses have limited time for infection control practices, such as hand hygiene and sterile procedures [12]. Furthermore, burnout and fatigue resulting from excessive workload can lead to decreased attentiveness and a higher likelihood of clinical errors. These findings underscore the urgent need for systematic workload assessments to optimize staffing and improve patient safety [2]. Despite global evidence on the importance of measuring nursing workload in ICUs, no study has been conducted in Nepal to assess nursing workload using the NAS tool. Given the staffing shortages, high patient burden, and limited resources in Nepalese hospitals, understanding ICU workload is critical for improving care quality and patient outcomes. The lack of objective workload assessments makes it difficult to identify staffing needs, optimize workforce distribution, and develop strategies to enhance patient safety [8]. The increasing demand for intensive care services in LMICs like Nepal, combined with nurse staffing shortages and excessive workload, highlights the need for systematic workload measurement tools. The NAS is a reliable and validated tool that can provide critical insights into ICU nursing workload, allowing for better resource allocation and workforce planning. Given the lack of research on NAS application in Nepal, this study is expected to fill an important knowledge gap by providing data-driven insights into ICU nursing workload and its impact on patient outcomes. By implementing evidence-based staffing models, hospitals can improve ICU efficiency, nurse well-being, and overall patient safety. This study aims to assess the nursing workload in Nepalese ICUs using the NAS and evaluate its predictive ability for patient outcomes. By analyzing the relationship between NAS scores and ICU mortality rates, this research seeks to provide evidence-based recommendations for staffing optimization and improving patient care in resource-limited settings.

## 2. Materials and methods

### Ethics statement

The study was approved by the Lincoln University College Research Ethics Committee (Ref: LUC/REC/INT/111/2022), the Nepal Health Research Council (Ref: NHRC/071/2022), and the Institutional Review Committee of Chitwan Medical College Teaching Hospital (Ref: CMC-IRC/078/079-150). Written informed consent was obtained from all participants or their legally authorized representatives (LARs) before enrolment. Participant confidentiality and privacy were maintained throughout the study, and all data were anonymized prior to analysis.

### Study design and setting

This prospective observational study was conducted to assess nursing workload using the Nursing Activities Score (NAS) and evaluate its predictive ability for patient outcomes among critically ill adults admitted to the intensive care units (ICUs) of a tertiary teaching hospital in a low-middle-income country (LMIC). The study was carried out in the Adult Medical ICU (20 beds) and Adult Surgical ICU (20 beds) of Chitwan Medical College Teaching Hospital, Nepal, where critically ill patients receive continuous multidisciplinary care. Daily ICU rounds were conducted by Critical Care Physicians (DM in Critical Care Medicine), supported by physiotherapists and ancillary staff. Ancillary staff assisted registered nurses with non-nursing activities but were not directly involved in patient nursing care. The ICUs followed standardized treatment and nursing care protocols. Although nurse-to-patient ratios varied according to patient acuity, staffing was generally predetermined and frequently insufficient to meet the nursing workload demands.

### Participants

Adult patients (≥18 years) admitted to either ICU between 07 March 2022 and 27 February 2023 were eligible for inclusion. Patients who required continuous nursing care and remained in the ICU for at least 24 hours were included. Patients who died or were discharged within 24 hours of ICU admission, were transferred to or from another hospital unit within the first 24 hours, or had incomplete NAS data were excluded. No pediatric patients were admitted to or included in this study.

A total of 501 consecutive eligible patients were recruited using convenience sampling. The sample size was determined based on previous ICU nursing workload studies and statistical considerations to ensure adequate power for evaluating the predictive performance of NAS.

### Nursing workforce and NAS implementation

ICU nursing care was provided by a mix of Registered Nurses (RNs) holding Proficiency Certificate Level (PCL) Nursing, Bachelor of Science (BSc) Nursing, and Bachelor of Nursing Science (BNS) qualifications. Most bedside nurses were PCL Nursing graduates who had completed short-term critical care training. ICU Nursing Supervisors possessed postgraduate qualifications in nursing with critical care training. During the study period, the Nursing Activities Score (NAS) was not routinely implemented in the study ICUs and was introduced for the first time as part of this research. Based on the findings of this study, the institution has continued routine NAS implementation to support nursing workload assessment and staffing decisions.

### Data collection and study variables

Primary data were collected prospectively using a structured paper-based data collection proforma attached to each patient’s nursing record. The institution used paper-based nursing and medical records throughout the study period.

The Nursing Activities Score (NAS) was assessed by trained bedside critical care nurses once during each nursing shift and was supervised daily by ICU Nursing Supervisors and the principal researcher. Before commencement of the study, nursing personnel received orientation regarding standardized NAS assessment procedures to ensure consistency in data collection. Daily NAS scores were compiled and verified by the Nursing Supervisors and researcher to obtain each patient’s 24-hour NAS score.

In addition to information extracted from nursing and medical records, direct patient assessment was performed whenever required and documented using the structured NAS proforma.

The NAS instrument consists of 23 nursing interventions, each assigned a weighted score according to the estimated nursing time required. The total NAS score ranges from 0 to 177%, representing the percentage of one nurse’s working time required to care for a single patient during a 24-hour period. Higher NAS scores indicate greater nursing workload and resource utilization.

Patient demographic characteristics, diagnostic categories, ICU length of stay, and ICU survival outcomes were also collected. The primary outcome variable was ICU survival status, categorized as survived or died at ICU discharge.

### Statistical analysis

Data were entered and analyzed using IBM SPSS Statistics version 25.0 (IBM Corp., Armonk, NY, USA). Continuous variables were assessed for normality using the Kolmogorov–Smirnov and Shapiro–Wilk tests and were found to be non-normally distributed. Therefore, continuous variables were summarized as median and interquartile range (IQR), whereas categorical variables were presented as frequencies and percentages.

The predictive performance of NAS for ICU survival outcome was evaluated using Receiver Operating Characteristic (ROC) curve analysis, and the Area Under the ROC Curve (AUROC), optimal cutoff value, sensitivity, specificity, and 95% confidence intervals were calculated. Model calibration was assessed using the Hosmer–Lemeshow goodness-of-fit test, with a *p*-value >0.05 indicating adequate model fit.

The association between NAS and ICU mortality was examined using binary logistic regression analysis, where NAS score was entered as the continuous independent variable, and ICU survival status (survived vs. died) served as the binary dependent variable. Odds ratios (Exp(B)) with 95% confidence intervals were reported. Differences in NAS scores between survivors and non-survivors were compared using the Mann–Whitney U test. Statistical significance was considered at p < 0.05.

## 3. Results

A total of 501 critically ill adult patients admitted to the Medical and Surgical Intensive Care Units (ICUs) were included in the study. The median age was 48.96 years (IQR: 30–65), with ages ranging from 18 to 86 years. More than half of the patients (54.3%) were aged over 50 years. Of the total participants, 306 (61.1%) were male and 195 (38.9%) were female. The most common primary diagnostic category was chronic obstructive pulmonary disease (COPD) and respiratory disorders (39.7%), followed by sepsis, septic shock and systemic infections (16.0%), neurological disorders (9.6%), and malignancy with post-operative complications (8.0%). The median ICU length of stay was 6 days (IQR: 5–8 days), ranging from 3 to 18 days. At ICU discharge, 316 (63.1%) patients survived, whereas 185 (36.9%) did not survive (Table 1).

**Table 1:** Demographic profile and clinical characteristics of critically ill patients (n = 501).

Variables	Category	Frequency (n)	Percentage (%)
Demographic Characteristics			
Age (years) Median: 48.96 years (IQR: 30–65) Range: 18–86	18–25	83	16.5
	25–35	65	13.0
	35–50	81	16.2
	Above 50	272	54.3
Sex	Male	306	61.1
	Female	195	38.9
Primary Diagnostic Categories			
Diagnosis	COPD and respiratory disorders	199	39.7
	Sepsis, septic shock & systemic infections	80	16.0
	Neurological disorders	48	9.6
	Malignancy & post-operative complications	40	8.0
	Renal disorders	36	7.2
	Poisoning & toxicological conditions	28	5.6
	Metabolic & autoimmune disorders	28	5.6
	Trauma & surgical emergencies	22	4.3
	Others	20	4.0
ICU Stay and Survival Outcome			
Length of ICU stay (days)	Median: 6 (IQR: 5–8); Range: 3–18	–	–
Survival outcome	Survived	316	63.1
	Non-survived	185	36.9
	Total	501	100.0

**Fig 1:**
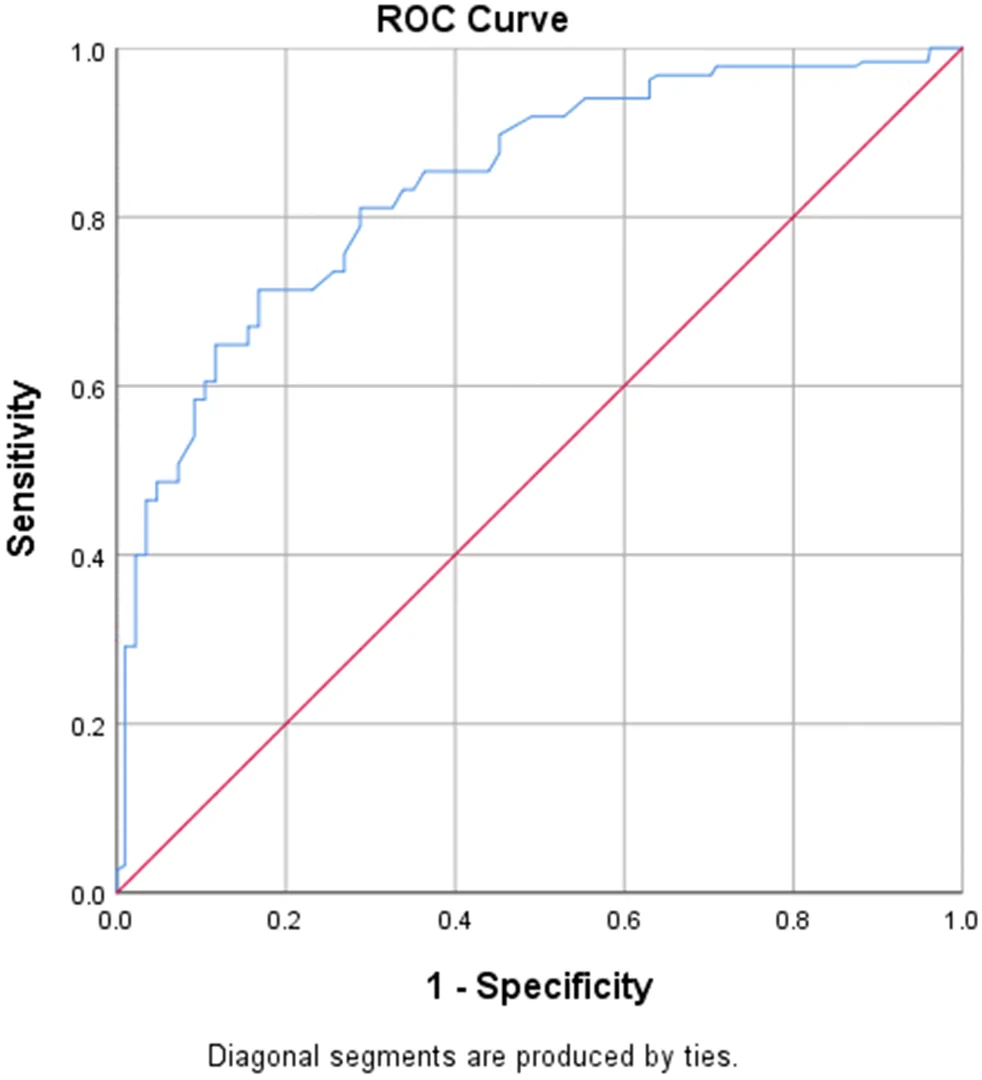
Receiver operating characteristic (ROC) curve for the Nursing Activities Score (NAS) predicting patient outcomes.

The predictive performance of the Nursing Activities Score (NAS) for ICU survival outcome was evaluated using receiver operating characteristic (ROC) curve analysis. The area under the receiver operating characteristic curve (AUROC) was 0.838 (95% CI: 0.802–0.875; *p* < 0.001). The optimal NAS cut-off value was 90.40, with a sensitivity of 73.5% and a specificity of 73.1% (Table 2 and Fig 1).

**Table 2:** Receiver Operating Characteristic curve (ROC) analysis for the Nursing Activities Score (NAS) scoring system for outcome of critically ill patients.

Scoring System	Cut off point	AUROC	p value	Sensitivity	Specificity	95% CI	
**Lower**	**Upper**
NAS	90.40	0.838	<0.001	0.735%	0.731%	.802	.875

The calibration of the NAS prediction model was assessed using the Hosmer–Lemeshow goodness-of-fit test. The model demonstrated a chi-square value of 8.967 with a p-value of 0.422, indicating an adequate model fit (Table 3).

**Table 3:** The Hosmer-Lemeshow test for the goodness-of-fit evaluation for Nursing Activities Score (NAS).

Scoring System	H-L Chi-square	p-value
NAS	8.967	0.422

Binary logistic regression analysis demonstrated a statistically significant association between NAS and ICU survival outcome. The regression coefficient (B) was −0.065, with an odds ratio [Exp(B)] of 0.937 (95% CI: 0.926–0.948; *p* < 0.001) (Table 4).

**Table 4:** Predictive performance assessment for the NAS scoring system.

Scoring system	B	Exp (B) Odds ratio	Significance p	95% CI for EXP(B)	
**Lower**	**Upper**
NAS	-.065	.937	<0.001	.926	.948

Comparison of NAS scores between survivors and non-survivors showed a statistically significant difference. The median NAS score was 110.70 among non-survivors and 76.20 among survivors. The difference was statistically significant (Mann–Whitney U = 9464.00; p < 0.001) (Table 5).

**Table 5:** Comparison of median Nursing Activities Score (NAS) among survivors and non-survivors.

Scoring system	Number	Median	Mean Rank	Mann-Whitney U	p-value
**NAS**				9464.00	<0.001
Non-survivor	185	110.70	357.84		
Survivor	316	76.20	188.45		

## 4. Discussion

This study assessed nursing workload in an ICU setting using the Nursing Activities Score (NAS) and evaluated its predictive value for patient outcomes. The findings revealed that higher NAS scores were associated with increased mortality risk, suggesting a strong correlation between workload, patient severity, and survival rates. These results align with previous studies highlighting the impact of nursing workload on patient care quality, ICU efficiency, and nurse well-being [1,10,13].

### Nursing workload assessment using NAS

The median NAS score in this study was 83.40 (IQR: 101.10–68.30, range: 39.2–134.4), indicating high nursing workload in the ICU. This aligns with findings by Miranda et al. (2003), who reported that NAS scores exceeding 80% indicate an excessive workload, often requiring additional staffing and resource allocation [11]. Similar studies in ICUs across high-income countries have reported comparable NAS scores, reinforcing the global concern regarding ICU nursing workload [9,12,14].

High NAS scores indicate substantial nursing care requirements, particularly in critically ill patients who need continuous monitoring, invasive procedures, and mechanical ventilation. Alhosani MI et al. (2023) found that ICU patients requiring mechanical ventilation have significantly higher NAS scores, which aligns with our study’s findings [15]. The higher NAS scores in non-survivors suggest that critically ill patients require more intensive nursing care, a pattern consistent with studies linking workload intensity with disease severity [10,14].

### Predictive value of NAS for patient outcomes

The Receiver Operating Characteristic (ROC) analysis demonstrated that NAS has strong predictive power for ICU patient outcomes, with an AUROC of 0.838 (p < 0.001). This suggests that NAS is an effective tool for identifying high-risk patients. The cut-off NAS score of 90.40, with 73.5% sensitivity and 73.1% specificity, indicates that patients requiring a NAS above this threshold are more likely to experience adverse outcomes. Similar findings have been reported in studies where higher NAS scores correlated with ICU mortality [14,16].

In a study by Nassiff A et al. (2018), NAS was found to be more accurate than traditional severity scoring systems, such as APACHE II and SOFA, in predicting mortality, reinforcing its value in ICU settings [17]. These findings support the argument that NAS should be integrated into ICU patient monitoring systems to improve early risk identification and workload distribution among nurses.

### Model fit and reliability of NAS

The Hosmer-Lemeshow goodness-of-fit test (Chi-square = 8.967, p = 0.422) confirmed that the NAS model fits well with observed ICU patient outcomes. A well-calibrated model strengthens the case for integrating NAS into ICU management strategies, as demonstrated in previous studies advocating for data-driven nursing workforce planning [3,18].

Similar findings were reported by Sardo PMG et al. (2023), where NAS demonstrated strong predictive accuracy across different ICU populations [16]. The strong statistical reliability of NAS suggests that it could be used as a workload monitoring tool, enabling hospitals to improve staff allocation, enhance patient safety, and reduce ICU strain.

### Association between NAS and mortality

The logistic regression analysis revealed a negative B coefficient (-0.065) and an odds ratio (Exp(B) = 0.937, p < 0.001), suggesting that higher NAS scores are linked to a lower probability of survival. This contradicts the assumption that higher nursing workload leads to better outcomes, instead emphasizing that increased NAS scores reflect greater patient severity rather than improved survival chances [4,9,19].

The comparison of NAS between survivors and non-survivors further supports this finding. Non-survivors had a median NAS of 110.70, significantly higher than survivors (76.20, p < 0.001). This is consistent with studies demonstrating that higher nursing workload is associated with critically ill patients who have a higher risk of mortality [10,12]. The Mann-Whitney U test results (p < 0.001) confirm a significant difference in workload between survivors and non-survivors, reinforcing the notion that ICU patients requiring intensive nursing care are at greater risk of mortality.

### Implications for ICU staffing and nursing workload management

The findings of this study highlight the urgent need for workforce optimization in ICUs, particularly in low-middle-income countries (LMICs) like Nepal. The high NAS scores indicate a substantial nursing workload, emphasizing the necessity of maintaining an adequate nurse-to-patient ratio [7]. Studies have shown that higher nurse staffing levels lead to better patient outcomes and reduced ICU mortality rates [1,4].

Additionally, Ghabi AAH et al. (2024) found that increasing the number of registered nurses per shift significantly reduced adverse events, highlighting the need for evidence-based staffing policies [13]. Future studies should evaluate real-time workload balancing using NAS scores, as proposed by Hasselgård AM et al. (2024), to improve ICU efficiency and patient safety [14].

This study confirms that NAS is a reliable tool for measuring nursing workload and predicting ICU patient outcomes. Higher NAS scores were associated with increased mortality risk, reflecting greater patient severity and resource needs. The findings highlight the critical role of adequate staffing in ICUs, emphasizing the need for data-driven workforce planning to balance workload distribution and optimize patient care.

By integrating NAS-based workload assessments into ICU staffing strategies, hospitals can enhance nurse efficiency, reduce burnout, and improve patient survival rates. Future research should explore multi-center validation of NAS and its application in diverse ICU settings, particularly in low-resource environments, to develop evidence-based staffing models for critical care.

### Strengths, limitations, and nursing implications

This study has notable strengths. It is the first to systematically assess nursing workload in intensive care units in Nepal using the Nursing Activities Score (NAS), thereby addressing a significant gap in local and regional evidence. The relatively large sample of critically ill patients and the application of robust statistical analyses, including ROC curve, logistic regression, and model fit testing, provide strong validity to the findings. By comparing survivors and non-survivors, the study demonstrates the direct clinical relevance of nursing workload to patient outcomes in resource-limited ICU settings.

However, several limitations should be acknowledged. Conducting the study in a single teaching hospital limits generalizability to other hospitals and regions. As an observational design, the study cannot establish causality between workload and patient outcomes. Reliance on NAS alone without incorporating other workload measures (e.g., nurse burnout, skill mix, or organizational factors) may not fully capture the complexity of ICU care. In addition, data were collected during particular period of time and may not reflect workload variations across shifts or seasonal patterns. Unmeasured confounders, such as physician practices and availability of resources, may also have influenced patient outcomes.

These findings have important clinical and policy implications. Future research should expand to multi-center and longitudinal designs, incorporating broader measures of workload and contextual factors. From a practical standpoint, NAS could be adopted as a routine monitoring and workforce planning tool to identify high-risk patients and allocate nursing resources more effectively. Improving nurse-to-patient ratios, guided by NAS assessments, may reduce preventable adverse events, improve patient safety, and promote sustainable working conditions for nurses in intensive care settings of low- and middle-income countries.

### Recommendations

Based on the findings of this study, the following recommendations are proposed:

1. Routine implementation of the Nursing Activities Score (NAS) in intensive care units is recommended to objectively assess nursing workload, facilitate evidence-based staffing decisions, and optimize the allocation of nursing resources.
2. ICU nurse staffing should be aligned with patients’ nursing workload, rather than relying solely on fixed nurse-to-patient ratios, to ensure safe, efficient, and high-quality critical care.
3. Hospital administrators and policymakers should consider integrating NAS into routine ICU nursing practice and workforce planning to support effective workload management and improve patient care.
4. Training and capacity-building programs should be conducted to enhance nurses’ competency in the standardized assessment and application of the Nursing Activities Score in routine clinical practice.
5. Multi-center studies involving different hospital settings and larger populations are recommended to further validate the applicability and generalizability of the Nursing Activities Score in Nepal and other low- and middle-income countries.
6. Future research should evaluate the relationship between NAS, nurse-to-patient ratios, nurse staffing levels, patient severity, and clinical outcomes, as well as examine the predictive contribution of individual NAS components to patient outcomes.
7. Longitudinal and implementation studies are recommended to assess the impact of routine NAS utilization on nursing workload management, resource allocation, patient outcomes, staff satisfaction, and healthcare costs.

## 5. Conclusion

The study assessed nursing workload and the utility of the Nursing Activities Score (NAS) in predicting outcomes for critically ill ICU patients. The results demonstrate that patients who did not survive required substantially more nursing care, as reflected by higher NAS scores. The NAS tool effectively distinguished between survivors and non-survivors, indicating its strength as a predictor of patient outcomes. Model evaluation confirmed that NAS provides a reliable fit for outcome prediction, and further analysis affirmed that increases in nursing workload were closely associated with a greater likelihood of mortality. These findings underscore the importance of using NAS not only to monitor nursing workload but also to identify patients at higher risk of poor outcomes. Overall, the study supports the clinical relevance of NAS as a practical and informative tool for guiding nurse staffing decisions and enhancing patient care in intensive care settings.

## Supporting information

S1 Data Study dataset used for the statistical analyses of the Nursing Activities Score (NAS) and patient outcomes among critically ill patients. (PDF)

S1 Text Global inclusivity questionnaire completed in accordance with PLOS Global Public Health reporting requirements. (DOCX)

## Abbreviations

AUROC: Area Under Receiver Operating Characteristics Curve
IQR: Interquartile range
LUC: Lincoln University College
REC: Research Ethics Committee
INT: International
CMC-IRC: Chitwan Medical College-Institutional Review Committee
Sens.: Sensitivity
Spec.: Specificity
L: Lower
U: Upper
CI: Confidence Interval
H-L: Hosmer-Lemeshow test

## References

[1] Aiken LH, Sloane DM, Bruyneel L, Van den Heede K, Griffiths P, Busse R, et al. Nurse staffing and education and hospital mortality in nine European countries: a retrospective observational study. Lancet. 2014;383(9931):1824–30. doi: 10.1016/S0140-6736(13)62631-8 24581683 PMC4035380

[2] Park J, Zhong X, Dong Y, Barwise A, Pickering BW. Investigating the cognitive capacity constraints of an ICU care team using a systems engineering approach. BMC Anesthesiol. 2022;22(1):10. doi: 10.1186/s12871-021-01548-7 34983402 PMC8724599

[3] Carayon P, Wetterneck TB, Rivera-Rodriguez AJ, Hundt AS, Hoonakker P, Holden R, et al. Human factors systems approach to healthcare quality and patient safety. Appl Ergon. 2014;45(1):14–25. doi: 10.1016/j.apergo.2013.04.023 23845724 PMC3795965

[4] Kane RL, Shamliyan TA, Mueller C, Duval S, Wilt TJ. The association of registered nurse staffing levels and patient outcomes: systematic review and meta-analysis. Med Care. 2007;45(12):1195–204. doi: 10.1097/MLR.0b013e3181468ca3 18007170

[5] Kartik M, Gopal PBN, Amte R. Quality indicators compliance survey in indian intensive care units. Indian J Crit Care Med. 2017;21(4):187–91. doi: 10.4103/ijccm.IJCCM_164_15 28515601 PMC5416784

[6] Patil SJ, Ambulkar R, Kulkarni AP. Patient safety in intensive care unit: what can we do better? Indian J Crit Care Med. 2023;27(3):163–5. doi: 10.5005/jp-journals-10071-24415 36960106 PMC10028712

[7] WHO. State of the world’s nursing: Investing in education, jobs, and leadership. WHO Press; 2021. Available from: https://www.who.int/publications/i/item/9789240003279

[8] Thapa DR, Subedi M, Ekström-Bergström A, Areskoug Josefsson K, Krettek A. Facilitators for and barriers to nurses’ work-related health-a qualitative study. BMC Nurs. 2022;21(1):218. doi: 10.1186/s12912-022-01003-z 35931988 PMC9356503

[9] West E, Barron DN, Harrison D, Rafferty AM, Rowan K, Sanderson C. Nurse staffing, medical staffing and mortality in intensive care: an observational study. Int J Nurs Stud. 2014;51(5):781–94. doi: 10.1016/j.ijnurstu.2014.02.007 24636667

[10] Numata Y, Schulzer M, van der Wal R, Globerman J, Semeniuk P, Balka E, et al. Nurse staffing levels and hospital mortality in critical care settings: literature review and meta-analysis. J Adv Nurs. 2006;55(4):435–48. doi: 10.1111/j.1365-2648.2006.03941.x 16866839

[11] Miranda DR, Nap R, de Rijk A, Schaufeli W, Iapichino G, TISS Working Group. Therapeutic Intervention Scoring System. Nursing activities score. Crit Care Med. 2003;31(2):374–82. doi: 10.1097/01.CCM.0000045567.78801.CC 12576939

[12] Oliveira AC de, Garcia PC, Nogueira L de S. Nursing workload and occurrence of adverse events in intensive care: a systematic review. Rev Esc Enferm USP. 2016;50(4):683–94. doi: 10.1590/S0080-623420160000500020 27680056

[13] Ghabi AAH, Ajaly HEM, Ojayli MA, Shbaly HA, Muharraq NE, Kenani KM, et al. Exploring the impact of nurse workload on patient safety outcomes in intensive care units: a multicenter study. J Int Crisis Risk Commun Res. 2024;6:496–503. doi: 10.63278/jicrcr.vi.773

[14] Hasselgård A-M, Stafseth SK, Kirkevold Ø. Nursing workload prediction for upcoming shifts: a retrospective observational exploratory study in the postoperative and intensive care unit. J Nurs Manag. 2024;2024:9703289. doi: 10.1155/2024/9703289 40224756 PMC11919223

[15] Alhosani MI, Ahmed FR, Al-Yateem N, Mobarak HS, AbuRuz ME. Assessment of nursing workload and adverse events reporting among critical care nurses in the United Arab Emirates. Open Nurs J. 2023;17:e187443462815. doi: 10.2174/0118744346281511231120054125

[16] Sardo PMG, Macedo RPA, Alvarelhão JJM, Simões JFL, Guedes JAD, Simões CJ, et al. Nursing workload assessment in an intensive care unit: a retrospective observational study using the Nursing Activities Score. Nurs Crit Care. 2023;28(2):288–97. doi: 10.1111/nicc.12854 36336353

[17] Nassiff A, Ricci de Araújo T, Menegueti MG, Bellissimo-Rodrigues F, Basile-Filho A, Laus AM. Nursing workload and patient mortality at an intensive care unit. Texto Contexto Enferm. 2018;27(4):e0390017. doi: 10.1590/0104-07072018000390017

[18] Gonçalves LA, Padilha KG, Cardoso Sousa RM. Nursing activities score (NAS): a proposal for practical application in intensive care units. Intensive Crit Care Nurs. 2007;23(6):355–61. doi: 10.1016/j.iccn.2007.04.009 17689247

[19] Vahedian-Azimi A, Hajiesmaeili M, Kangasniemi M, Fornés-Vives J, Hunsucker RL, Rahimibashar F, et al. Effects of stress on critical care nurses: a national cross-sectional study. J Intensive Care Med. 2019;34(4):311–22. doi: 10.1177/0885066617696853 29277137

