# Supplementary material for: Performance of Nursing Activities Score in intensive care units of a teaching hospital in a low-middle-income country: An observational study
Source: PLOS Glob Public Health. 2026 Aug 24;6(8):e0006169. doi: 10.1371/journal.pgph.0006169 (PMC13502596; doi:10.1371/journal.pgph.0006169)
Supplement: Supplementary file 1 [file pgph.0006169.s001.pdf]

S.N.	Age	Sex	LOS -ICU	NAS	Outcome
1	81	1	13	130	0
2	35	2	5	61.4	1
3	21	1	7	83.2	1
4	68	1	10	100.9	0
5	20	2	3	71.9	1
6	52	2	9	101.1	0
7	24	1	11	116.2	0
8	40	1	15	101.1	1
9	18	1	6	49	1
10	32	1	10	112.3	0
11	65	1	12	119.1	0
12	78	1	7	91.9	0
13	50	1	5	75.8	1
14	45	2	3	43.7	1
15	50	1	6	91.7	1
16	22	2	8	122.7	0
17	20	1	5	61.8	1
18	75	1	7	127.4	0
19	75	1	9	131.2	0
20	60	2	13	111.7	1
21	58	2	8	46.7	1
22	70	2	12	131.3	0
23	49	1	8	89.6	1
24	50	1	6	52.1	1
25	48	2	4	69.5	1
26	70	2	5	72.9	1
27	68	1	8	116.8	0
28	78	1	6	75.8	1
29	30	2	5	80.8	1
30	46	1	4	59.4	1
31	24	2	6	91.3	1
32	73	1	10	101.3	0
33	55	1	4	43.5	1
34	73	2	6	82.5	1
35	44	2	8	99.1	1
36	52	1	5	72.9	1
37	26	1	3	62.1	1
38	33	1	6	46.8	1
39	80	2	13	121.8	0
40	65	2	5	77.8	1
41	85	2	7	93.2	1
42	30	2	5	77.8	1
43	19	1	6	83.2	0
44	22	2	8	80.8	1
45	65	1	6	92.7	1
46	86	1	4	77.8	0

47	24	2	5	72.9	0
48	45	1	3	77.8	1
49	60	2	9	113.7	0
50	55	1	5	62.8	1
51	18	1	4	50.8	1
52	54	2	4	68.2	1
53	18	2	3	55.3	1
54	26	2	6	83.9	1
55	62	2	12	111.3	1
56	56	1	10	116.1	1
57	67	2	11	89.3	0
58	72	2	8	91.7	1
59	29	1	5	67.3	1
60	50	1	6	48.3	1
61	45	2	8	68.3	0
62	35	2	5	81.9	1
63	50	1	6	76.2	1
64	75	1	6	49.2	1
65	62	1	10	89.6	0
66	25	1	8	91.1	1
67	55	1	12	91.9	0
68	73	1	8	72.5	1
69	18	1	5	59.7	1
70	30	1	6	98.7	0
71	45	1	8	89.2	1
72	54	1	9	114.2	0
73	23	1	8	96.5	0
74	22	1	3	48.7	1
75	50	1	6	97.1	1
76	50	2	4	77.9	0
77	73	1	4	79.3	1
78	70	1	7	92.1	1
79	27	2	5	60.1	1
80	65	1	6	116.2	0
81	44	1	4	52.5	1
82	20	1	4	67.2	1
83	18	2	7	113.6	1
84	60	1	5	71.4	1
85	22	2	5	62.4	1
86	48	1	8	91.3	0
87	65	1	6	56.1	1
88	37	1	5	121	1
89	72	1	7	78.2	1
90	57	1	8	121.3	0
91	70	2	9	110.7	0
92	37	1	6	82.9	1
93	73	2	7	99.3	0

94	18	2	6	72.1	1
95	65	1	6	91.4	1
96	47	1	12	128.6	0
97	18	2	5	51.3	1
98	82	1	10	96.1	0
99	26	2	8	72.7	1
100	52	1	8	89.6	0
101	54	1	4	55.2	1
102	60	2	12	117.5	0
103	71	2	16	126.3	0
104	25	2	6	91.3	1
105	76	2	8	83.4	1
106	62	1	7	78.2	0
107	48	1	6	43.5	1
108	45	1	8	89.7	0
109	65	1	6	78.9	1
110	30	2	4	66.5	1
111	35	2	7	91.7	1
112	30	1	11	82.5	0
113	27	2	8	92.5	0
114	35	1	5	82.3	1
115	50	1	6	91.7	1
116	78	1	12	113.9	0
117	75	1	8	121.7	0
118	18	2	3	39.2	1
119	65	1	3	56.7	1
120	50	2	4	61.9	1
121	62	1	7	130.2	0
122	50	1	8	122.8	0
123	73	1	13	134.4	0
124	47	1	7	92.9	1
125	67	2	10	130.6	0
126	81	1	13	130	0
127	35	2	5	61.4	1
128	21	1	7	83.2	1
129	68	1	10	100.9	0
130	20	2	3	71.9	1
131	52	2	9	101.1	0
132	24	1	11	116.2	0
133	40	1	15	101.1	1
134	18	1	6	49	1
135	32	1	10	112.3	0
136	65	1	12	119.1	0
137	78	1	7	91.9	0
138	50	1	5	75.8	1
139	71	1	3	43.7	0
140	50	1	6	91.7	1

141	22	2	8	122.7	0
142	20	1	5	61.8	1
143	75	1	7	127.4	0
144	75	1	9	131.2	0
145	60	2	13	111.7	1
146	58	2	8	46.7	1
147	28	2	12	131.3	1
148	27	2	8	89.6	0
149	50	1	6	52.1	1
150	48	2	4	69.5	1
151	70	2	5	72.9	1
152	68	1	8	116.8	0
153	78	1	6	75.8	1
154	30	2	5	80.8	1
155	46	1	4	59.4	1
156	24	2	6	91.3	1
157	45	1	10	101.3	0
158	55	1	4	43.5	1
159	73	2	6	82.5	1
160	44	2	8	99.1	1
161	52	1	5	72.9	1
162	26	1	3	62.1	1
163	33	1	6	46.8	1
164	80	2	13	121.8	0
165	65	2	5	77.8	1
166	85	2	7	93.2	1
167	30	2	5	77.8	1
168	19	1	6	83.2	0
169	22	2	8	80.8	1
170	65	1	6	92.7	1
171	86	1	4	77.8	0
172	24	2	5	72.9	0
173	45	1	3	77.8	1
174	60	2	9	113.7	0
175	55	1	5	62.8	1
176	18	1	4	50.8	1
177	54	2	4	68.2	1
178	18	2	3	55.3	1
179	26	2	6	83.9	1
180	62	2	12	111.3	1
181	56	1	10	116.1	1
182	67	2	11	89.3	0
183	72	2	8	91.7	1
184	29	1	5	67.3	1
185	50	1	6	48.3	1
186	45	2	8	68.3	0
187	35	2	5	81.9	1

188	50	1	6	76.2	1
189	75	1	6	49.2	1
190	30	2	10	89.6	1
191	25	1	8	91.1	1
192	55	1	12	91.9	0
193	73	1	8	72.5	1
194	18	1	5	59.7	1
195	30	1	6	98.7	0
196	45	1	8	89.2	1
197	54	1	9	114.2	0
198	23	1	8	96.5	0
199	22	1	3	48.7	1
200	50	1	6	97.1	1
201	50	2	4	77.9	0
202	73	1	4	79.3	1
203	70	1	7	92.1	1
204	27	2	5	60.1	1
205	65	1	6	116.2	0
206	44	1	4	52.5	1
207	20	1	4	67.2	1
208	18	2	7	113.6	1
209	60	1	5	71.4	1
210	22	2	5	62.4	1
211	48	1	8	91.3	0
212	65	1	6	56.1	1
213	37	1	5	121	1
214	72	1	7	78.2	1
215	57	1	8	121.3	0
216	70	2	9	110.7	0
217	37	1	6	82.9	1
218	73	2	7	99.3	0
219	18	2	6	72.1	1
220	65	1	6	91.4	1
221	47	1	12	128.6	0
222	18	2	5	51.3	1
223	82	1	10	96.1	0
224	26	2	8	72.7	1
225	52	1	8	89.6	0
226	54	1	4	55.2	1
227	60	2	12	117.5	0
228	71	2	16	126.3	0
229	25	2	6	91.3	1
230	76	2	8	83.4	1
231	62	1	7	78.2	0
232	48	1	6	43.5	1
233	45	1	8	89.7	0
234	65	1	6	78.9	1

235	30	2	4	66.5	1
236	35	2	7	91.7	1
237	30	1	11	82.5	0
238	27	2	8	92.5	0
239	35	1	5	82.3	1
240	50	1	6	91.7	1
241	78	1	12	113.9	0
242	75	1	8	121.7	0
243	18	2	3	39.2	1
244	65	1	3	56.7	1
245	50	2	4	61.9	1
246	62	1	7	130.2	0
247	50	1	8	122.8	0
248	73	1	13	134.4	0
249	47	1	7	92.9	1
250	67	2	10	130.6	0
251	81	1	13	130	0
252	35	2	5	61.4	1
253	21	1	7	83.2	1
254	68	1	10	100.9	0
255	20	2	3	71.9	1
256	52	2	9	101.1	0
257	24	1	11	116.2	0
258	40	1	15	101.1	1
259	18	1	6	49	1
260	32	1	10	112.3	0
261	65	1	12	119.1	0
262	78	1	7	91.9	0
263	50	1	5	75.8	1
264	65	1	3	43.7	0
265	50	1	6	91.7	1
266	22	2	8	122.7	0
267	20	1	5	61.8	1
268	75	1	7	127.4	0
269	75	1	9	131.2	0
270	60	2	13	111.7	1
271	58	2	8	46.7	1
272	28	2	12	131.3	1
273	49	1	8	89.6	1
274	50	1	6	52.1	1
275	48	2	4	69.5	1
276	70	2	5	72.9	1
277	68	1	8	116.8	0
278	78	1	6	75.8	1
279	30	2	5	80.8	1
280	46	1	4	59.4	1
281	24	2	6	91.3	1

282	60	1	10	101.3	1
283	55	1	4	43.5	1
284	73	2	6	82.5	1
285	44	2	8	99.1	1
286	52	1	5	72.9	1
287	72	2	3	62.1	0
288	33	1	6	46.8	1
289	80	2	13	121.8	0
290	65	2	5	77.8	1
291	85	2	7	93.2	1
292	30	2	5	77.8	1
293	19	1	6	83.2	0
294	22	2	8	80.8	1
295	65	1	6	92.7	1
296	86	1	4	77.8	0
297	24	2	5	72.9	0
298	45	1	3	77.8	1
299	60	2	9	113.7	0
300	55	1	5	62.8	1
301	18	1	4	50.8	1
302	67	2	4	68.2	0
303	18	2	3	55.3	1
304	26	2	6	83.9	1
305	62	2	12	111.3	1
306	56	1	10	116.1	1
307	67	2	11	89.3	0
308	72	2	8	91.7	1
309	29	1	5	67.3	1
310	50	1	6	48.3	1
311	45	2	8	68.3	0
312	35	2	5	81.9	1
313	50	1	6	76.2	1
314	75	1	6	49.2	1
315	30	2	10	89.6	1
316	25	1	8	91.1	1
317	55	1	12	91.9	0
318	73	1	8	72.5	1
319	18	1	5	59.7	1
320	30	1	6	98.7	0
321	45	1	8	89.2	1
322	54	1	9	114.2	0
323	23	1	8	96.5	0
324	22	1	3	48.7	1
325	50	1	6	97.1	1
326	50	2	4	77.9	0
327	73	1	4	79.3	1
328	70	1	7	92.1	1

329	27	2	5	60.1	1
330	65	1	6	116.2	0
331	44	1	4	52.5	1
332	20	1	4	67.2	1
333	18	2	7	113.6	1
334	60	1	5	71.4	1
335	22	2	5	62.4	1
336	48	1	8	91.3	0
337	65	1	6	56.1	1
338	37	1	5	121	1
339	72	1	7	78.2	1
340	57	1	8	121.3	0
341	70	2	9	110.7	0
342	37	1	6	82.9	1
343	73	2	7	99.3	0
344	18	2	6	72.1	1
345	65	1	6	91.4	1
346	47	1	12	128.6	0
347	18	2	5	51.3	1
348	82	1	10	96.1	0
349	26	2	8	72.7	1
350	52	1	8	89.6	0
351	54	1	4	55.2	1
352	60	2	12	117.5	0
353	71	2	16	126.3	0
354	25	2	6	91.3	1
355	76	2	8	83.4	1
356	62	1	7	78.2	0
357	48	1	6	43.5	1
358	45	1	8	89.7	0
359	65	1	6	78.9	1
360	30	2	4	66.5	1
361	35	2	7	91.7	1
362	30	1	11	82.5	0
363	27	2	8	92.5	0
364	35	1	5	82.3	1
365	50	1	6	91.7	1
366	78	1	12	113.9	0
367	75	1	8	121.7	0
368	18	2	3	39.2	1
369	65	1	3	56.7	1
370	50	2	4	61.9	1
371	62	1	7	130.2	0
372	50	1	8	122.8	0
373	73	1	13	134.4	0
374	47	1	7	92.9	1
375	67	2	10	130.6	0

376	81	1	13	130	0
377	35	2	5	61.4	1
378	21	1	7	83.2	1
379	68	1	10	100.9	0
380	20	2	3	71.9	1
381	52	2	9	101.1	0
382	24	1	11	116.2	0
383	40	1	15	101.1	1
384	18	1	6	49	1
385	32	1	10	112.3	0
386	65	1	12	119.1	0
387	78	1	7	91.9	0
388	50	1	5	75.8	1
389	71	1	3	43.7	0
390	50	1	6	91.7	1
391	22	2	8	122.7	0
392	20	1	5	61.8	1
393	75	1	7	127.4	0
394	75	1	9	131.2	0
395	60	2	13	111.7	1
396	58	2	8	46.7	1
397	28	2	12	131.3	1
398	49	1	8	89.6	1
399	50	1	6	52.1	1
400	48	2	4	69.5	1
401	70	2	5	72.9	1
402	68	1	8	116.8	0
403	78	1	6	75.8	1
404	30	2	5	80.8	1
405	46	1	4	59.4	1
406	24	2	6	91.3	1
407	60	1	10	101.3	1
408	55	1	4	43.5	1
409	73	2	6	82.5	1
410	44	2	8	99.1	1
411	52	1	5	72.9	1
412	57	1	3	62.1	0
413	33	1	6	46.8	1
414	80	2	13	121.8	0
415	65	2	5	77.8	1
416	85	2	7	93.2	1
417	30	2	5	77.8	1
418	19	1	6	83.2	0
419	22	2	8	80.8	1
420	65	1	6	92.7	1
421	86	1	4	77.8	0
422	24	2	5	72.9	0

423	45	1	3	77.8	1
424	60	2	9	113.7	0
425	55	1	5	62.8	1
426	52	2	4	50.8	0
427	54	2	4	68.2	1
428	18	2	3	55.3	1
429	26	2	6	83.9	1
430	62	2	12	111.3	1
431	56	1	10	116.1	1
432	67	2	11	89.3	0
433	72	2	8	91.7	1
434	29	1	5	67.3	1
435	50	1	6	48.3	1
436	45	2	8	68.3	0
437	35	2	5	81.9	1
438	50	1	6	76.2	1
439	75	1	6	49.2	1
440	30	2	10	89.6	1
441	25	1	8	91.1	1
442	55	1	12	91.9	0
443	73	1	8	72.5	1
444	18	1	5	59.7	1
445	30	1	6	98.7	0
446	45	1	8	89.2	1
447	54	1	9	114.2	0
448	23	1	8	96.5	0
449	22	1	3	48.7	1
450	50	1	6	97.1	1
451	50	2	4	77.9	0
452	73	1	4	79.3	1
453	70	1	7	92.1	1
454	27	2	5	60.1	1
455	65	1	6	116.2	0
456	44	1	4	52.5	1
457	20	1	4	67.2	1
458	18	2	7	113.6	1
459	60	1	5	71.4	1
460	22	2	5	62.4	1
461	48	1	8	91.3	0
462	65	1	6	56.1	1
463	37	1	5	121	1
464	72	1	7	78.2	1
465	57	1	8	121.3	0
466	70	2	9	110.7	0
467	37	1	6	82.9	1
468	73	2	7	99.3	0
469	18	2	6	72.1	1

470	65	1	6	91.4	1
471	47	1	12	128.6	0
472	18	2	5	51.3	1
473	82	1	10	96.1	0
474	26	2	8	72.7	1
475	52	1	8	89.6	0
476	54	1	4	55.2	1
477	60	2	12	117.5	0
478	71	2	16	126.3	0
479	25	2	6	91.3	1
480	76	2	8	83.4	1
481	62	1	7	78.2	0
482	48	1	6	43.5	1
483	45	1	8	89.7	0
484	65	1	6	78.9	1
485	30	2	4	66.5	1
486	35	2	7	91.7	1
487	30	1	11	82.5	0
488	27	2	8	92.5	0
489	35	1	5	82.3	1
490	50	1	6	91.7	1
491	78	1	12	113.9	0
492	75	1	8	121.7	0
493	18	2	3	39.2	1
494	65	1	3	56.7	1
495	50	2	4	61.9	1
496	62	1	7	130.2	0
497	50	1	8	122.8	0
498	73	1	13	134.4	0
499	47	1	7	92.9	1
500	67	2	10	130.6	0
501	61	1	18	132.1	0
