# Supplementary material for: Performance of Nursing Activities Score in intensive care units of a teaching hospital in a low-middle-income country: An observational study
Source: PLOS Glob Public Health. 2026 Aug 24;6(8):e0006169. doi: 10.1371/journal.pgph.0006169 (PMC13502596; doi:10.1371/journal.pgph.0006169)
Supplement: Supplementary file 3 [file pgph.0006169.s003.docx]

Review of manuscript PGPH-D-26-00826

«Performance of Nursing Activities Score in intensive care units of a teaching hospital in a low-middle-income country: An observational study.»

Thank you for the opportunity to read and come with suggestios for better understanding and clarity in the manuscript of the tool NAS. All over it is a nice work with ICU patients and workload.

Abstract: in the methods. Please add timeperiod for data collection.

1.Introduction

It is long, however a gap for using an objective tool in your country is obvious and great. And you place the study in low-midle-income countries, which is important for patients and nurses.

2. Materials and methods

Where there any children included or not treated in this unit? Please clarify. A question who did perform the NAS score? Nurses bedside, the researcher, one or several persons? Did they do the score per shift or once a day? Was it a paperbased system or online system? Or data from medical records? Was the NAS new? Implenting process? More over what personal both in numbers, education i.e take care of the patients? And how many beds has the unit and staffing? Please add.

In the part of statistical explanation in sentence Continous vaiables (NAS scores, age, ICU stay duration) were.... was not normal. Plase add normal distrubution. Later you say Logistic Regression Analysis was performed....however the variables you mention are conitious varaiables so why not linear regressions? Or if logistic how did you dichotomize?

Results: must be rewritten there are so many statements and comments, better to place in discussion where it belongs. The results must be presented neutral. One example are «This results indicates a high nursing workload in the ICU, suggesting that nurses are required to provide substantial care per patient....etc.

In the sentence «For Lenght of Stay (LOS), the median was 6 days, with IQR of 5 to 8 days, meaning that the midle 50% of patients had a hospital stay within this range. First median does not need to be explained, second is it ICU-stay or was it actually hospital stay? Please remove some of it and clarify.

Furthermore, an example The findings of ROC: «these findinds are comparable to other icu-based studies etc..» please move to discussion!

Discussion

In the second part under Nursing Workload Assessment Using NAS; the references 12,9, 14, please have them in ordered number 9, 12, 14. And later on under Association Between NAS and Mortality the references appear 9, 4, 19, please in order. If you add the suggestion from above (results) a revision will be needed.

Conclusion

It is well written.

Tables and figure.

I do miss to have table 1 with all patients data, age, sex, lenght of stay, diagnosis etc. At the moment, someare mentioned in text, some are missing. I suggest to remove table 2 it is easy to have in text (as you actually have). Table 3 must be extended and if this is the logistic regression (?) please add all relevant information for the readers.

A role in scientific writing if a table only have one row, «it is not a table». Reconsider the use of tables.
