# Supplementary material for: Performance of Nursing Activities Score in intensive care units of a teaching hospital in a low-middle-income country: An observational study
Source: PLOS Glob Public Health. 2026 Aug 24;6(8):e0006169. doi: 10.1371/journal.pgph.0006169 (PMC13502596; doi:10.1371/journal.pgph.0006169)
Supplement: Supplementary file 4 [file pgph.0006169.s004.docx]

The topic is relevant within the context of care in intensive care units, particularly regarding critical care and nursing workforce allocation. However, it is considered that the study still lacks clarification on certain aspects that require further elaboration.

In the Methods section, I noted the absence of a clear description of the research protocol. Was data collection conducted in a general Intensive Care Unit (ICU)? How many beds does the studied ICU have? Additionally, the data collection process itself is not sufficiently detailed—what were the sources of the data?

It is also important to clarify whether the institution where the study was conducted uses paper-based or electronic medical records. How many individuals were responsible for data collection? Was there any prior training to standardize the retrieval of patient information?

Furthermore, it is unclear whether the data were extracted exclusively from medical records or if any direct patient assessment was required. Lastly, it is not clear whether the Nursing Activities Score (NAS) is routinely used in the studied ICU.

In the Discussion section, it would be valuable to further explore the notion that higher Nursing Activities Score (NAS) values do not necessarily indicate greater clinical severity. Elevated NAS scores may, for instance, be associated with chronically ill but clinically stable patients who require a high level of nursing care related to feeding, hygiene, and mobility, as well as the maintenance of artificial airways, such as in patients with tracheostomies.

Additionally, if all 23 items comprising the NAS were collected, it is recommended to present and discuss the results of these items in detail, in order to better justify the scores obtained and enhance the interpretation of the findings.
