# Supplementary material for: Performance of Nursing Activities Score in intensive care units of a teaching hospital in a low-middle-income country: An observational study
Source: PLOS Glob Public Health. 2026 Aug 24;6(8):e0006169. doi: 10.1371/journal.pgph.0006169 (PMC13502596; doi:10.1371/journal.pgph.0006169)
Supplement: Supplementary file 5 [file pgph.0006169.s005.docx]

**Response to the Editor and Reviewers**

**Manuscript ID:** PGPH-D-26-00826
**Title:** *Performance of Nursing Activities Score in Intensive Care Units of a Teaching Hospital in a Low-Middle-Income Country: An Observational Study*

Dear Dr. Jen Edwards and the Reviewers,

On behalf of all co-authors, we would like to express our sincere gratitude to you and the four reviewers for your careful evaluation of our manuscript and for the thoughtful, constructive, and insightful comments provided during the review process. We greatly appreciate the time and expertise invested in reviewing our work.

We have carefully considered every comment and suggestion raised by the Editor and reviewers. In response, we have thoroughly revised the manuscript to improve its scientific rigor, methodological clarity, presentation, and overall quality. The revisions include, but are not limited to:

- Revision of the Abstract, including the study period.
- Updated the Introduction and strengthened the international context while retaining relevant seminal references.
- Reorganization of the Methods section into clearly defined subsections with expanded descriptions of the study setting, participants, data collection procedures, ICU characteristics, staffing, NAS implementation, and statistical analysis.
- Revision of the Results section to present findings objectively by removing interpretative statements and improving table presentation.
- Strengthening of the Discussion by reducing duplication, improving interpretation of findings, adding practical implications, and addressing reviewer concerns regarding the Nursing Activities Score (NAS).
- Revision of the Conclusion and improvement of the overall language, grammar, and manuscript readability.
- Updating of the Data Availability Statement, Financial Disclosure, Inclusivity in Global Research Questionnaire, and submission of separate TIFF figure files in accordance with the journal's requirements.
- Revision of tables, including consolidation of demographic and clinical characteristics into a single table and updating the regression table as suggested.

We believe that these revisions have substantially strengthened the manuscript and have addressed all concerns raised by the Editor and reviewers. A detailed point-by-point response to every comment is provided in the following pages, with corresponding revisions incorporated into the revised manuscript.

We sincerely thank the Editor and reviewers once again for their valuable comments, which have significantly improved the quality of our manuscript. We hope that the revised version is now suitable for publication in **PLOS Global Public Health**.

Thank you for your time and consideration.

Yours sincerely,

**Prof. Raj Kumar Mehta**
**Corresponding Author**
Department of Critical Care Nursing
Chitwan Medical College Teaching Hospital
Bharatpur, Chitwan, Nepal

**Point-by-Point Response to the Editor and Reviewers**

We sincerely thank the Editor and all reviewers for their valuable comments and suggestions. Each comment has been carefully considered, and the manuscript has been revised accordingly. Below, we provide a detailed point-by-point response to every issue raised.

**Section I. Response to the Editor**

**Journal Requirements**

1. Please include a complete copy of PLOS’ questionnaire on inclusivity in global research in your revised manuscript. Our policy for research in this area aims to improve transparency in the reporting of research performed outside of researchers’ own country or community. The policy applies to researchers who have travelled to a different country to conduct research, research with Indigenous populations or their lands, and research on cultural artefacts. The questionnaire can also be requested at the journal’s discretion for any other submissions, even if these conditions are not met.  Please find more information on the policy and a link to download a blank copy of the questionnaire here: . Please upload a completed version of your questionnaire as Supporting Information when you resubmit your manuscript.

***Response:***

*Thank you very much for your valuable feedback. A completed copy of the PLOS Questionnaire on Inclusivity in Global Research has been prepared and uploaded as Supporting Information with the revised manuscript, in accordance with the journal's requirements.*

2. Please amend your detailed Financial Disclosure statement. This is published with the article. It must therefore be completed in full sentences and contain the exact wording you wish to be published.

a. Please clarify all sources of funding (financial or material support) for your study. List the grants (with grant number) or organizations (with url) that supported your study, including funding received from your institution. 
b. State the initials, alongside each funding source, of each author to receive each grant.
c. State what role the funders took in the study. If the funders had no role in your study, please state: “The funders had no role in study design, data collection and analysis, decision to publish, or preparation of the manuscript.”
d. If any authors received a salary from any of your funders, please state which authors and which funders.

If you did not receive any funding for this study, please simply state: “The authors received no specific funding for this work.”

***Response:***

*Thank you very much for your valuable feedback. The Financial Disclosure statement has been revised in accordance with the journal's requirements. As this study did not receive any specific financial support, the statement has been updated as follows:*

*"The authors received no specific funding for this work."*

3. Please provide separate figure files in .tif or .eps format.

For more information about figure files please see our guidelines:  LINK

https://journals.plos.org/globalpublichealth/s/figures 
https://journals.plos.org/globalpublichealth/s/figures#loc-file-requirements

***Response:***

*Thank you very much for your valuable feedback. Separate figure files in* ***.tif*** *format have been prepared and uploaded with the revised submission in accordance with the journal's figure file requirements.*

4. In the online submission form, you indicated that "The data supporting the findings of this article are available from the corresponding author upon reasonable request.".

All PLOS journals now require all data underlying the findings described in their manuscript to be freely available to other researchers, either 
1. In a public repository, 
2. Within the manuscript itself, or 
3. Uploaded as supplementary information.

This policy applies to all data except where public deposition would breach compliance with the protocol approved by your research ethics board. If your data cannot be made publicly available for ethical or legal reasons (e.g., public availability would compromise patient privacy), please explain your reasons by return email and your exemption request will be escalated to the editor for approval. Your exemption request will be handled independently and will not hold up the peer review process, but will need to be resolved should your manuscript be accepted for publication. One of the Editorial team will then be in touch if there are any issues.

***Response:***

*Thank you very much for your valuable feedback. The Data Availability Statement has been revised in accordance with the journal's requirements and updated as follows:*

*"The data supporting the findings of this article are available within the manuscript itself."*

If the reviewer comments include a recommendation to cite specific previously published works, please review and evaluate these publications to determine whether they are relevant and should be cited. There is no requirement to cite these works unless the editor has indicated otherwise. 

Additional Editor Comments (if provided):

[Note: HTML markup is below. Please do not edit.]

**Reviewers' comments:**

Reviewer's Responses to Questions

**Comments to the Author**

1. Does this manuscript meet PLOS Global Public Health’s [publication criteria](https://journals.plos.org/globalpublichealth/s/criteria-for-publication)? Is the manuscript technically sound, and do the data support the conclusions? The manuscript must describe methodologically and ethically rigorous research with conclusions that are appropriately drawn based on the data presented.

Reviewer #1: Yes

Reviewer #2: Yes

Reviewer #3: Yes

Reviewer #4: Partly

***Response to Reviewers:***

*We sincerely thank the Editor and all four reviewers for their thorough evaluation of our manuscript and for their constructive, insightful, and valuable comments. We greatly appreciate the time and effort invested in reviewing our work. We have carefully considered all comments and have revised the manuscript accordingly. We believe that these revisions have substantially improved the clarity, methodological rigor, and overall quality of the manuscript. A detailed, point-by-point response to each comment is provided below*.

2. Has the statistical analysis been performed appropriately and rigorously?

Reviewer #1: No

Reviewer #2: Yes

Reviewer #3: Yes

Reviewer #4: Yes

***Response:***

*We sincerely thank the reviewers for their valuable feedback. We have carefully revised the statistical analysis, addressed all relevant comments, and clarified the analytical methods in the revised manuscript to improve its rigor and transparency.*

3. Have the authors made all data underlying the findings in their manuscript fully available (please refer to the Data Availability Statement at the start of the manuscript PDF file)?

The [PLOS Data policy](https://journals.plos.org/globalpublichealth/s/data-availability) requires authors to make all data underlying the findings described in their manuscript fully available without restriction, with rare exception. The data should be provided as part of the manuscript or its supporting information, or deposited to a public repository. For example, in addition to summary statistics, the data points behind means, medians and variance measures should be available. If there are restrictions on publicly sharing data—e.g. participant privacy or use of data from a third party—those must be specified.

Reviewer #1: No

Reviewer #2: Yes

Reviewer #3: Yes

Reviewer #4: Yes

***Response:***

*We sincerely thank the reviewers for their valuable feedback. The Data Availability Statement has been revised in accordance with the journal's policy. All data underlying the findings are now available within the manuscript itself.*

4. Is the manuscript presented in an intelligible fashion and written in standard English?

PLOS Global Public Health does not copyedit accepted manuscripts, so the language in submitted articles must be clear, correct, and unambiguous. Any typographical or grammatical errors should be corrected at revision, so please note any specific errors here.

Reviewer #1: Yes

Reviewer #2: Yes

Reviewer #3: Yes

Reviewer #4: Yes

***Response:***

*We sincerely thank all the reviewers for their positive assessment of the manuscript's language and presentation. We have carefully proofread the revised manuscript and made minor language and grammatical refinements to further improve its clarity, readability, and overall quality*.

5. Review Comments to the Author

Please use the space provided to explain your answers to the questions above. You may also include additional comments for the author, including concerns about dual publication, research ethics, or publication ethics. (Please upload your review as an attachment if it exceeds 20,000 characters)

**Reviewer #1: Review of manuscript PGPH-D-26-00826**
«Performance of Nursing Activities Score in intensive care units of a teaching hospital in a low-middle-income country: An observational study. »

Thank you for the opportunity to read and come with suggestions for better understanding and clarity in the manuscript of the tool NAS. All over it is a nice work with ICU patients and workload.
***Response:***

*We sincerely thank the reviewer for the thoughtful, constructive, and encouraging comments. We greatly appreciate your positive assessment of our work and your valuable suggestions, which have helped improve the clarity and quality of the manuscript.*

**Abstract:** in the methods. Please add time period for data collection.

***Response:***

*We sincerely thank the reviewer for this valuable suggestion. The Methods section of the abstract has been revised accordingly. The following sentence has been added:*

*"Participants were recruited over a one-year period, from 07 March 2022 to 27 February 2023."*

1.Introduction
It is long, however a gap for using an objective tool in your country is obvious and great. And you place the study in low-middle-income countries, which is important for patients and nurses.

***Response:***

*We sincerely thank the reviewer for the positive and encouraging comments. We greatly appreciate your recognition of the significance and relevance of our study, particularly the importance of using an objective nursing workload assessment tool in a low-middle-income country setting.*

2. Materials and methods
Where there any children included or not treated in this unit? Please clarify. A question who did perform the NAS score? Nurses’ bedside, the researcher, one or several persons? Did they do the score per shift or once a day? Was it a paper-based system or online system? Or data from medical records? Was the NAS new? Implementing process? More over what personal both in numbers, education i.e. take care of the patients? And how many beds has the unit and staffing? Please add.
In the part of statistical explanation in sentence Continuous variables (NAS scores, age, ICU stay duration) were.... was not normal. Please add normal distribution. Later you say Logistic Regression Analysis was performed.... however, the variables you mention are continuous variables so why not linear regressions? Or if logistic, how did you dichotomize?

***Response:***

*We sincerely thank the reviewer for the valuable and constructive comments. The manuscript has been revised accordingly.*

1. *We have clarified that no children were included in this study, as participants were recruited exclusively from the Adult ICUs (Medical ICU and Surgical ICU).*
2. *We have clarified that the Nursing Activities Score (NAS) was assessed by bedside critical care nurses and supervised by the ICU Nursing Supervisors and the researcher. NAS was recorded once per shift using a paper-based structured proforma attached to each patient's nursing record. Daily NAS scores were compiled and verified by the ICU Nursing Supervisors and the researcher.*
3. *We have added that the NAS was newly introduced in the study ICUs as part of the implementation process.*
4. *We have provided additional information on the ICU settings and staffing. The Medical ICU (MICU) has 20 beds and the Surgical ICU (SICU) has 20 beds. The MICU is staffed by 20 nurses, with five nurses per shift across three shifts per day. The SICU is staffed by 26 nurses, with six nurses per shift across three shifts per day. Nursing staff included approximately equal proportions of PCL Nursing and BSc Nursing graduates.*
5. *The statistical methods section has been revised to clarify that the continuous variables were assessed for normality and were found to be non-normally distributed. Therefore, non-parametric summary statistics (median and interquartile range) were used. Logistic regression analysis was appropriately performed because the independent variable (NAS score) was continuous, while the dependent variable was ICU survival status, a binary outcome categorized as survived or died at ICU discharge.*

Results: must be rewritten there are so many statements and comments, better to place in discussion where it belongs. The results must be presented neutral. One example are «This results indicates a high nursing workload in the ICU, suggesting that nurses are required to provide substantial care per patient....etc.
In the sentence «For Lenght of Stay (LOS), the median was 6 days, with IQR of 5 to 8 days, meaning that the midle 50% of patients had a hospital stay within this range. First median does not need to be explained, second is it ICU-stay or was it actually hospital stay? Please remove some of it and clarify.
Furthermore, an example The findings of ROC: «these findinds are comparable to other icu-based studies etc..» please move to discussion!
***Response:***

*We sincerely thank the reviewer for these valuable suggestions. The Results section has been thoroughly revised to present the findings in a neutral manner. Interpretative statements and comparisons with previous studies have been removed from the Results and incorporated into the Discussion where appropriate. We have also clarified that the reported length of stay refers to ICU stay and revised the text accordingly.*

Discussion
In the second part under Nursing Workload Assessment Using NAS; the references 12,9, 14, please have them in ordered number 9, 12, 14. And later on under Association Between NAS and Mortality the references appear 9, 4, 19, please in order. If you add the suggestion from above (results) a revision will be needed.
***Response:***

*We sincerely thank the reviewer for this valuable suggestion. The reference citations in the Discussion section have been revised and reordered in sequential numerical order according to the journal's referencing style. All suggested corrections have been addressed in the revised manuscript.*

Conclusion
It is well written.

***Response:***

*We sincerely thank the reviewer for the positive and encouraging comment. We greatly appreciate your recognition of the quality of our conclusion.*

Tables and figure.

I do miss to have table 1 with all patients data, age, sex, lenght of stay, diagnosis etc. At the moment, someare mentioned in text, some are missing. I suggest to remove table 2 it is easy to have in text (as you actually have). Table 3 must be extended and if this is the logistic regression (?) please add all relevant information for the readers.
A role in scientific writing if a table only have one row, «it is not a table». Reconsider the use of tables.

***Response:***

*We sincerely thank the reviewer for these valuable suggestions. The recommendations have been implemented in the revised manuscript. Table 1 has been updated to include the demographic and clinical characteristics of the study participants for improved clarity and completeness.*

**Reviewer #2: Thank you for submitting to PLOS Global Public Health Journal.**
The authors have presented a study exploring the Nursing Activities Score- a validated and psychometrically tested score utilized in many high-income countries to determine resources and nurse to patient ratios in ICUs. The score has not been extensively tested in low-income countries, and this is the first study in Nepal in a large ICU setting adding to the body of knowledge already known about the NAS.
The authors used a large sample size- convenience sampling- that was determined prior to the study and used a prospective observational design for their research method. The authors used ROC method to determine the NAS predictive ability. The authors collected the data over a year 2022-2023 and the results of the study demonstrated a ROC of 0.838 showing that the NAS had a high predictive value for determining the workload of the nurses. Logistic regression analysis showed that higher NAS scores were significantly associated with increased mortality risk (Exp(B) = 0.937, p < 0.001). Non-survivors had significantly higher NAS scores (110.70) compared with survivors (76.20, p < 0.001).
The authors concluded that the NAS is a reliable tool for assessing ICU nursing workload and predicting patient outcomes. Higher NAS scores reflect greater patient severity and increased risk of mortality, highlighting the importance of optimized staffing and workload management in ICU settings.

Strengths of the study:
1. First study to explore NAS in Nepal in the ICU setting
2. Robust statistical analysis used
3. Authors had no missing data due to the study design
4. Inclusion and exclusion criteria clearly defined
5. Sample size calculated prior to the study

Limitations to consider:
1. One ICU only was used in the study
2. The time period used was still in the COVID pandemic timeframe as the WHO officially declared COVID -19 had ended in May 2023
3. Prospective observational design using convenience sampling
4. Varying nurse to patient ratios is a confounder
***Response:***

*We sincerely thank the reviewer for the thorough, thoughtful, and encouraging evaluation of our manuscript. We greatly appreciate your recognition of the novelty, methodological rigor, and potential contribution of our study to the evidence on the Nursing Activities Score in a low-middle-income country setting. Your positive assessment is highly valued.*

**Please consider answering the following inquires I have:**
1. The authors stated that the nurse-to-patient ratio was varying depending on severity of patients etc- how did this confounder get accounted for in the analysis? and would it have affected the NAS score?

***Response:***

*We sincerely thank the reviewer for this insightful comment. This study primarily established the baseline Nursing Activities Score (NAS) in our ICU setting. Although the nurse-to-patient ratio was generally predetermined, it was often inadequate relative to patient severity and nursing workload. We acknowledge that this could have acted as a potential confounder; however, it was not evaluated in the present study. We appreciate this important suggestion and will incorporate nurse-to-patient ratio and other potential confounding factors into future studies.*

2. Can you please in a few sentences explain the education of RNs in Nepal and is the mixture always RNs in the ICU and what post graduate training or training in general do they go through to work in the ICU?

***Response:***

*We sincerely thank the reviewer for this valuable question. In Nepal, Registered Nurses are qualified through either a 3-year Proficiency Certificate Level (PCL) Nursing program, a 4-year Bachelor of Science (BSc) Nursing program, or a 3-year Bachelor of Nursing Science (BNS) program following PCL Nursing. ICU nursing staff typically comprise a mix of these qualified nurses, with most holding a PCL Nursing qualification and receiving additional short-term critical care training. ICU nursing supervisors generally hold a Master of Nursing (MN) in Adult Health Nursing with critical care training. In recent years, MN in Critical Care Nursing (Critical Care Nurse Specialist, CCNS) programs have been introduced in three teaching hospitals in Nepal, including our institution, with the first cohort having already graduated. In addition, short-term critical care training programs are regularly conducted by the Critical Care Nurses Association of Nepal (CCNAN) and teaching hospitals.*

3. The authors mentioned confounders such as resource availability and MDs etc- please state if the following is available as routine in ICUs in Nepal:
1. Respiratory Therapists
2. Ancillary staff that help the RN at the bedside
3. Do the ICU MDs round each day
4. Do many Nepalese nurses go to other countries to work- do you use a lot of agency staff?
Thank you for considering these as these will affect the NAS indirectly and many readers would also like to know more about the healthcare in Nepal.

***Response:***

*We sincerely thank the reviewer for these insightful questions. We have clarified these points in the revised manuscript. In our ICU, physiotherapists are available as part of the multidisciplinary team, although there are no dedicated respiratory therapists. Ancillary staff are available during all shifts to support Registered Nurses (RNs) with non-nursing tasks; however, they are not directly involved in providing nursing care to patients. Critical Care Physicians (DM in Critical Care Medicine) conduct daily ICU rounds. We also acknowledge that many Nepalese nurses migrate to work abroad, resulting in a high nursing turnover across the country. However, agency nursing staff are not used in our institution; instead, vacant positions are filled through regular recruitment processes.*

Thank you for allowing my review and for writing the paper to include more countries in this research.

**Reviewer #3:** The Introduction section relies heavily on outdated literature. It would significantly strengthen the manuscript to incorporate more recent sources and position the study within the current international context. Expanding the contemporary relevance of the topic would improve the scientific value and impact of the paper.

***Response:***

*We sincerely thank the reviewer for this valuable suggestion. We carefully reviewed the literature and included the most relevant evidence available for the study topic, irrespective of publication date. Both recent and seminal studies have been cited where appropriate to provide a comprehensive scientific background and place our findings within the current body of evidence. We have also updated the Introduction with additional recent references where relevant to further strengthen the international context of the study.*

There is considerable duplication of content across sections. In particular, definitions, abbreviations, and explanatory descriptions introduced in the Introduction are repeated again in the Methodology section. This repetition should be reduced, and key terms should be defined once and used consistently throughout the manuscript.
***Response:***

*We sincerely thank the reviewer for this valuable suggestion. The issue has been addressed in the revised manuscript. Repetitive definitions, abbreviations, and explanatory descriptions have been removed, and key terms are now defined once and used consistently throughout the manuscript.*

The Methodology section is currently presented as one continuous block of text, which makes it difficult to follow. It is recommended to divide this section into clearly defined subsections, such as:
Study design
Participants / Sample
Instruments / Measures
Data collection
Statistical analysis
Ethical considerations
A more structured format would greatly improve readability and clarity.
***Response:***

*We sincerely thank the reviewer for this valuable suggestion. The Methodology section has been revised and reorganized into clearly defined subsections, including Study Design, Participants, Study Instrument, Data Collection, Statistical Analysis, and Ethical Considerations, to improve the clarity, readability, and overall structure of the manuscript.*

There is noticeable repetition between the Results and Discussion sections, with some results being restated rather than interpreted. The Results section should focus strictly on presenting findings, while the Discussion should interpret these findings, compare them with existing literature, and provide critical analysis. Reducing duplication will improve the overall quality of the manuscript.
***Response:***

*We sincerely thank the reviewer for this valuable suggestion. The issue has been appropriately addressed in the revised manuscript. The Results section now presents the findings objectively, while repetitive content has been removed and incorporated into the Discussion, which now focuses on the interpretation of findings, comparison with existing literature, and critical analysis.*

It is recommended to include a clearly defined subsection for practical implications or recommendations within the Discussion. This would enhance the applied value of the study and provide clearer guidance for clinical practice, policy, or future research.

***Response:***

*We sincerely thank the reviewer for this valuable suggestion. The recommendation has been addressed in the revised manuscript by incorporating a dedicated subsection on the practical implications and recommendations, highlighting the clinical relevance of the findings and directions for future research.*

**Reviewer #4:** The topic is relevant within the context of care in intensive care units, particularly regarding critical care and nursing workforce allocation. However, it is considered that the study still lacks clarification on certain aspects that require further elaboration.

In the Methods section, I noted the absence of a clear description of the research protocol. Was data collection conducted in a general Intensive Care Unit (ICU)? How many beds does the studied ICU have? Additionally, the data collection process itself is not sufficiently detailed—what were the sources of the data?

It is also important to clarify whether the institution where the study was conducted uses paper-based or electronic medical records. How many individuals were responsible for data collection? Was there any prior training to standardize the retrieval of patient information?
Furthermore, it is unclear whether the data were extracted exclusively from medical records or if any direct patient assessment was required. Lastly, it is not clear whether the Nursing Activities Score (NAS) is routinely used in the studied ICU.

In the Discussion section, it would be valuable to further explore the notion that higher Nursing Activities Score (NAS) values do not necessarily indicate greater clinical severity. Elevated NAS scores may, for instance, be associated with chronically ill but clinically stable patients who require a high level of nursing care related to feeding, hygiene, and mobility, as well as the maintenance of artificial airways, such as in patients with tracheostomies.
Additionally, if all 23 items comprising the NAS were collected, it is recommended to present and discuss the results of these items in detail, in order to better justify the scores obtained and enhance the interpretation of the findings.

***Reply for 1^st^ paragraph feedback:***

***Response:***

*We sincerely thank the reviewer for the valuable and constructive comments. We have carefully addressed all the concerns raised and revised the manuscript accordingly to improve its clarity, scientific rigor, and overall quality.*

***Reply for 2^nd^ paragraph feedback:***

***Response:***

*We sincerely thank the reviewer for this valuable comment. The Methods section has been revised to provide a clearer description of the research protocol. We have specified that the study was conducted in the Adult Medical ICU (20 beds) and Surgical ICU (20 beds). The data collection process has been described in greater detail, including that primary data were collected daily using a structured paper-based proforma by trained bedside nurses under the supervision of ICU Nursing Supervisors and the researcher. These revisions improve the clarity and reproducibility of the study methodology.*

***Reply for 3^rd^ paragraph feedback:***

***Response:***

*We sincerely thank the reviewer for these valuable comments. The Methods section has been revised to address these points. The study was conducted in ICUs that use paper-based nursing and medical records. Data were collected from the Adult Medical ICU (20 beds) and Surgical ICU (20 beds). Primary data were collected daily by trained bedside nurses, supervised by the ICU Nursing Supervisors and the researcher, following prior orientation to ensure standardized data collection. In addition to information extracted from patient records, direct patient assessment was performed and documented using a structured paper-based NAS proforma. We have also clarified that the Nursing Activities Score (NAS) was not routinely used in the study ICUs and was implemented for the first time as part of this research. Based on the findings of this study and its demonstrated utility, the researcher and the institution have continued the routine use of NAS in the ICU to support nursing workload assessment and staffing decisions.*

***Reply for 4^th^ paragraph feedback:***

***Response:***

*We sincerely thank the reviewer for these valuable suggestions. The Discussion has been revised to acknowledge that higher NAS scores do not necessarily reflect greater clinical severity but rather represent increased nursing workload and care demands, including the care required by chronically ill yet clinically stable patients.*

*Regarding the NAS items, all 23 NAS components were collected for each patient. However, for this study, the total NAS score was calculated and analyzed in accordance with the study objectives. Presenting and discussing each of the 23 individual items is beyond the scope of the current manuscript and would substantially increase its length. We appreciate this important suggestion and intend to explore and report the individual NAS item profiles in future publications.*

6. PLOS authors have the option to publish the peer review history of their article ([what does this mean?](https://journals.plos.org/globalpublichealth/s/editorial-and-peer-review-process#loc-peer-review-history)). If published, this will include your full peer review and any attached files.


**Do you want your identity to be public for this peer review?** If you choose “no”, your identity will remain anonymous but your review may still be made public.


For information about this choice, including consent withdrawal, please see our [Privacy Policy](https://www.plos.org/privacy-policy).

Reviewer #1: **Yes:** Siv Karlsson Stafseth

Reviewer #2: **Yes:** Elizabeth Scruth

Reviewer #3: **Yes:** Olga Cerela-Boltunova

Reviewer #4: **Yes:** Aline Affonso Luna

[NOTE: If reviewer comments were submitted as an attachment file, they will be attached to this email and accessible via the submission site. Please log into your account, locate the manuscript record, and check for the action link "View Attachments". If this link does not appear, there are no attachment files.]

**Figure Resubmissions:**

 While revising your submission, we strongly recommend that you use PLOS’s NAAS tool (https://ngplosjournals.pagemajik.ai/artanalysis) to test your figure files. NAAS can convert your figure files to the TIFF file type and meet basic requirements (such as print size, resolution), or provide you with a report on issues that do not meet our requirements and that NAAS cannot fix.

**After uploading your figures to PLOS’s NAAS tool -** https://ngplosjournals.pagemajik.ai/artanalysis, NAAS will process the files provided and display the results in the "Uploaded Files" section of the page as the processing is complete. If the uploaded figures meet our requirements (or NAAS is able to fix the files to meet our requirements), the figure will be marked as "fixed" above. If NAAS is unable to fix the files, a red "failed" label will appear above. When NAAS has confirmed that the figure files meet our requirements, please download the file via the download option, and include these NAAS processed figure files when submitting your revised manuscript.

*In compliance with data protection regulations, you may request that we remove your personal registration details at any time.* [*(Remove my information/details)*](https://www.editorialmanager.com/pgph/login.asp?a=r)*. Please contact the publication office if you have any questions.*
