# Supplementary material for: Performance of Nursing Activities Score in intensive care units of a teaching hospital in a low-middle-income country: An observational study
Source: PLOS Glob Public Health. 2026 Aug 24;6(8):e0006169. doi: 10.1371/journal.pgph.0006169 (PMC13502596; doi:10.1371/journal.pgph.0006169)
Supplement: Peer review history

# Peer review: Decision Letter 0

**DOI:** 10.1371/journal.pgph.0006169.r001

**Authors:** Jen Edwards

3 May 2026

PGPH-D-26-00826

Performance of Nursing Activities Score in intensive care units of a teaching hospital in a low-middle-income country: An observational study

PLOS Global Public Health

Dear Dr. Mehta,

Thank you for submitting your manuscript to PLOS Global Public Health. After careful consideration, we feel that it has merit but does not fully meet PLOS Global Public Health’s publication criteria as it currently stands. Therefore, we invite you to submit a revised version of the manuscript that addresses the points raised during the review process.

The manuscript has been evaluated by four reviewers, and their comments are available below. The reviewers have raised a number of major concerns. They feel the manuscript needs improvement in the methodology and depth of dicussion. They also have a number of queries regarding the statistical analysis. Could you please carefully revise the manuscript to address all comments raised?

Please submit your revised manuscript by Jun 30 2026 11:59PM. If you will need more time than this to complete your revisions, please reply to this message or contact the journal office at globalpubhealth@plos.org. When you're ready to submit your revision, log on to https://www.editorialmanager.com/pgph/ and select the 'Submissions Needing Revision' folder to locate your manuscript file.

Please include the following items when submitting your revised manuscript:

- A letter that responds to each point raised by the editor and reviewer(s). You should upload this letter as a separate file labeled 'Response to Reviewers'.
- A marked-up copy of your manuscript that highlights changes made to the original version. You should upload this as a separate file labeled 'Revised Manuscript with Track Changes'.
- An unmarked version of your revised paper without tracked changes. You should upload this as a separate file labeled 'Manuscript'.

Guidelines for resubmitting your figure files are available below the reviewer comments at the end of this letter.

As the corresponding author, your ORCID iD is verified in the submission system and will appear in the published article. PLOS supports the use of ORCID, and we encourage all coauthors to register for an ORCID iD and use it as well. Please encourage your coauthors to verify their ORCID iD within the submission system before final acceptance, as unverified ORCID iDs will not appear in the published article. *Only*  the individual author can complete the verification step; PLOS staff *cannot*  verify ORCID iDs on behalf of authors.

We look forward to receiving your revised manuscript.

Kind regards,

Jen Edwards

Staff Editor

PLOS Global Public Health

Journal Requirements:

1. Please include a complete copy of PLOS’ questionnaire on inclusivity in global research in your revised manuscript. Our policy for research in this area aims to improve transparency in the reporting of research performed outside of researchers’ own country or community. The policy applies to researchers who have travelled to a different country to conduct research, research with Indigenous populations or their lands, and research on cultural artefacts. The questionnaire can also be requested at the journal’s discretion for any other submissions, even if these conditions are not met.  Please find more information on the policy and a link to download a blank copy of the questionnaire here: https://journals.plos.org/globalpublichealth/s/best-practices-in-research-reporting. Please upload a completed version of your questionnaire as Supporting Information when you resubmit your manuscript.

2. Please amend your detailed Financial Disclosure statement. This is published with the article. It must therefore be completed in full sentences and contain the exact wording you wish to be published.

a. Please clarify all sources of funding (financial or material support) for your study. List the grants (with grant number) or organizations (with url) that supported your study, including funding received from your institution.

b. State the initials, alongside each funding source, of each author to receive each grant.

c. State what role the funders took in the study. If the funders had no role in your study, please state: “The funders had no role in study design, data collection and analysis, decision to publish, or preparation of the manuscript.”

d. If any authors received a salary from any of your funders, please state which authors and which funders.

If you did not receive any funding for this study, please simply state: “The authors received no specific funding for this work.”

3. Please provide separate figure files in .tif or .eps format.

For more information about figure files please see our guidelines:  LINK

https://journals.plos.org/globalpublichealth/s/figures

https://journals.plos.org/globalpublichealth/s/figures#loc-file-requirements

4. In the online submission form, you indicated that "The data supporting the findings of this article are available from the corresponding author upon reasonable request.".

All PLOS journals now require all data underlying the findings described in their manuscript to be freely available to other researchers, either

1. In a public repository,

2. Within the manuscript itself, or

3. Uploaded as supplementary information.

This policy applies to all data except where public deposition would breach compliance with the protocol approved by your research ethics board. If your data cannot be made publicly available for ethical or legal reasons (e.g., public availability would compromise patient privacy), please explain your reasons by return email and your exemption request will be escalated to the editor for approval. Your exemption request will be handled independently and will not hold up the peer review process, but will need to be resolved should your manuscript be accepted for publication. One of the Editorial team will then be in touch if there are any issues.

If the reviewer comments include a recommendation to cite specific previously published works, please review and evaluate these publications to determine whether they are relevant and should be cited. There is no requirement to cite these works unless the editor has indicated otherwise.

Additional Editor Comments (if provided):

[Note: HTML markup is below. Please do not edit.]

Reviewers' comments:

Reviewer's Responses to Questions

**Comments to the Author**

1. Does this manuscript meet PLOS Global Public Health’s publication criteria?

Reviewer #1: Yes

Reviewer #2: Yes

Reviewer #3: Yes

Reviewer #4: Partly

**********

2. Has the statistical analysis been performed appropriately and rigorously?

Reviewer #1: No

Reviewer #2: Yes

Reviewer #3: Yes

Reviewer #4: Yes

**********

3. Have the authors made all data underlying the findings in their manuscript fully available (please refer to the Data Availability Statement at the start of the manuscript PDF file)?

The PLOS Data policy

Reviewer #1: No

Reviewer #2: Yes

Reviewer #3: Yes

Reviewer #4: Yes

**********

4. Is the manuscript presented in an intelligible fashion and written in standard English?

Reviewer #1: Yes

Reviewer #2: Yes

Reviewer #3: Yes

Reviewer #4: Yes

**********

Reviewer #1: Review of manuscript PGPH-D-26-00826

«Performance of Nursing Activities Score in intensive care units of a teaching hospital in a low-middle-income country: An observational study.»

Thank you for the opportunity to read and come with suggestios for better understanding and clarity in the manuscript of the tool NAS. All over it is a nice work with ICU patients and workload.

Abstract: in the methods. Please add timeperiod for data collection.

1.Introduction

It is long, however a gap for using an objective tool in your country is obvious and great. And you place the study in low-midle-income countries, which is important for patients and nurses.

2. Materials and methods

Where there any children included or not treated in this unit? Please clarify. A question who did perform the NAS score? Nurses bedside, the researcher, one or several persons? Did they do the score per shift or once a day? Was it a paperbased system or online system? Or data from medical records? Was the NAS new? Implenting process? More over what personal both in numbers, education i.e take care of the patients? And how many beds has the unit and staffing? Please add.

In the part of statistical explanation in sentence Continous vaiables (NAS scores, age, ICU stay duration) were.... was not normal. Plase add normal distrubution. Later you say Logistic Regression Analysis was performed....however the variables you mention are conitious varaiables so why not linear regressions? Or if logistic how did you dichotomize?

Results: must be rewritten there are so many statements and comments, better to place in discussion where it belongs. The results must be presented neutral. One example are «This results indicates a high nursing workload in the ICU, suggesting that nurses are required to provide substantial care per patient....etc.

In the sentence «For Lenght of Stay (LOS), the median was 6 days, with IQR of 5 to 8 days, meaning that the midle 50% of patients had a hospital stay within this range. First median does not need to be explained, second is it ICU-stay or was it actually hospital stay? Please remove some of it and clarify.

Furthermore, an example The findings of ROC: «these findinds are comparable to other icu-based studies etc..» please move to discussion!

Discussion

In the second part under Nursing Workload Assessment Using NAS; the references 12,9, 14, please have them in ordered number 9, 12, 14. And later on under Association Between NAS and Mortality the references appear 9, 4, 19, please in order. If you add the suggestion from above (results) a revision will be needed.

Conclusion

It is well written.

Tables and figure.

I do miss to have table 1 with all patients data, age, sex, lenght of stay, diagnosis etc. At the moment, someare mentioned in text, some are missing. I suggest to remove table 2 it is easy to have in text (as you actually have). Table 3 must be extended and if this is the logistic regression (?) please add all relevant information for the readers.

A role in scientific writing if a table only have one row, «it is not a table». Reconsider the use of tables.

Reviewer #2: Thank you for submitting to PLOS Global Public Health Journal.

The authors have presented a study exploring the Nursing Activities Score- a validated and psychometrically tested score utilized in many high-income countries to determine resources and nurse to patient ratios in ICUs. The score has not been extensively tested in low-income countries, and this is the first study in Nepal in a large ICU setting adding to the body of knowledge already known about the NAS.

The authors used a large sample size- convenience sampling- that was determined prior to the study and used a prospective observational design for their research method. The authors used ROC method to determine the NAS predictive ability. The authors collected the data over a year 2022-2023 and the results of the study demonstrated a ROC of 0.838 showing that the NAS had a high predictive value for determining the workload of the nurses. Logistic regression analysis showed that higher NAS scores were significantly associated with increased mortality risk (Exp(B) = 0.937, p < 0.001). Non-survivors had significantly higher NAS scores (110.70) compared with survivors (76.20, p < 0.001).

The authors concluded that the NAS is a reliable tool for assessing ICU nursing workload and predicting patient outcomes. Higher NAS scores reflect greater patient severity and increased risk of mortality, highlighting the importance of optimized staffing and workload management in ICU settings.

Strengths of the study:

1. First study to explore NAS in Nepal in the ICU setting

2. Robust statistical analysis used

3. Authors had no missing data due to the study design

4. Inclusion and exclusion criteria clearly defined

5. Sample size calculated prior to the study

Limitations to consider:

1. One ICU only was used in the study

2. The time period used was still in the COVID pandemic timeframe as the WHO officially declared COVID -19 had ended in May 2023

3. Prospective observational design using convenience sampling

4. Varying nurse to patient ratios is a confounder

Please consider answering the following inquires I have:

1. The authors stated that the nurse to patient ratio was varying depending on severity of patients etc- how did this confounder get accounted for in the analysis? and would it have affected the NAS score?

2. Can you please in a few sentences explain the education of RNs in Nepal and is the mixture always RNs in the ICU and what post graduate training or training in general do they go through to work in the ICU?

3. The authors mentioned confounders such as resource availability and MDs etc- please state if the following is available as routine in ICUs in Nepal:

1. Respiratory Therapists

2. Ancillary staff that help the RN at the bedside

3. Do the ICU MDs round each day

4. Do many Nepalese nurses go to other countries to work- do you use a lot of agency staff?

Thank you for considering these as these will affect the NAS indirectly and many readers would also like to know more about the healthcare in Nepal.

Thank you for allowing my review and for writing the paper to include more countries in this research.

Reviewer #3: The Introduction section relies heavily on outdated literature. It would significantly strengthen the manuscript to incorporate more recent sources and position the study within the current international context. Expanding the contemporary relevance of the topic would improve the scientific value and impact of the paper.

There is considerable duplication of content across sections. In particular, definitions, abbreviations, and explanatory descriptions introduced in the Introduction are repeated again in the Methodology section. This repetition should be reduced, and key terms should be defined once and used consistently throughout the manuscript.

The Methodology section is currently presented as one continuous block of text, which makes it difficult to follow. It is recommended to divide this section into clearly defined subsections, such as:

Study design

Participants / Sample

Instruments / Measures

Data collection

Statistical analysis

Ethical considerations

A more structured format would greatly improve readability and clarity.

There is noticeable repetition between the Results and Discussion sections, with some results being restated rather than interpreted. The Results section should focus strictly on presenting findings, while the Discussion should interpret these findings, compare them with existing literature, and provide critical analysis. Reducing duplication will improve the overall quality of the manuscript.

It is recommended to include a clearly defined subsection for practical implications or recommendations within the Discussion. This would enhance the applied value of the study and provide clearer guidance for clinical practice, policy, or future research.

Reviewer #4: The topic is relevant within the context of care in intensive care units, particularly regarding critical care and nursing workforce allocation. However, it is considered that the study still lacks clarification on certain aspects that require further elaboration.

In the Methods section, I noted the absence of a clear description of the research protocol. Was data collection conducted in a general Intensive Care Unit (ICU)? How many beds does the studied ICU have? Additionally, the data collection process itself is not sufficiently detailed—what were the sources of the data?

It is also important to clarify whether the institution where the study was conducted uses paper-based or electronic medical records. How many individuals were responsible for data collection? Was there any prior training to standardize the retrieval of patient information?

Furthermore, it is unclear whether the data were extracted exclusively from medical records or if any direct patient assessment was required. Lastly, it is not clear whether the Nursing Activities Score (NAS) is routinely used in the studied ICU.

In the Discussion section, it would be valuable to further explore the notion that higher Nursing Activities Score (NAS) values do not necessarily indicate greater clinical severity. Elevated NAS scores may, for instance, be associated with chronically ill but clinically stable patients who require a high level of nursing care related to feeding, hygiene, and mobility, as well as the maintenance of artificial airways, such as in patients with tracheostomies.

Additionally, if all 23 items comprising the NAS were collected, it is recommended to present and discuss the results of these items in detail, in order to better justify the scores obtained and enhance the interpretation of the findings.

**********

what does this mean?). If published, this will include your full peer review and any attached files.

**Do you want your identity to be public for this peer review?**  If you choose “no”, your identity will remain anonymous but your review may still be made public.

For information about this choice, including consent withdrawal, please see our Privacy Policy

Reviewer #1: **Yes:**  Siv Karlsson Stafseth

Reviewer #2: **Yes:**  Elizabeth Scruth

Reviewer #3: **Yes:**  Olga Cerela-Boltunova

Reviewer #4: **Yes:**  Aline Affonso Luna

**********

[NOTE: If reviewer comments were submitted as an attachment file, they will be attached to this email and accessible via the submission site. Please log into your account, locate the manuscript record, and check for the action link "View Attachments". If this link does not appear, there are no attachment files.]

Attachment Submitted filename: Review of manuscript PGPH.docx

Attachment Submitted filename: Avaliation PLOS.docx

# Author comment: Author response to Decision Letter 1

**DOI:** 10.1371/journal.pgph.0006169.r002

16 Jul 2026

Attachment Submitted filename: Response to Reviewers.docx

# Peer review: Decision Letter 1

**DOI:** 10.1371/journal.pgph.0006169.r003

**Authors:** Andrés F. Henao-Martínez

4 Aug 2026

Performance of Nursing Activities Score in intensive care units of a teaching hospital in a low-middle-income country: An observational study

PGPH-D-26-00826R1

Dear Professor Mehta,

We are pleased to inform you that your manuscript 'Performance of Nursing Activities Score in intensive care units of a teaching hospital in a low-middle-income country: An observational study' has been provisionally accepted for publication in PLOS Global Public Health.

Before your manuscript can be formally accepted you will need to complete some formatting changes, which you will receive in a follow up email. A member of our team will be in touch with a set of requests.

Please note that your manuscript will not be scheduled for publication until you have made the required changes, so a swift response is appreciated.

IMPORTANT: The editorial review process is now complete. PLOS will only permit corrections to spelling, formatting or significant scientific errors from this point onwards. Requests for major changes, or any which affect the scientific understanding of your work, will cause delays to the publication date of your manuscript.

If your institution or institutions have a press office, please notify them about your upcoming paper to help maximize its impact. If they'll be preparing press materials, please inform our press team as soon as possible -- no later than 48 hours after receiving the formal acceptance. Your manuscript will remain under strict press embargo until 2 pm Eastern Time on the date of publication. For more information, please contact globalpubhealth@plos.org.

Thank you again for supporting Open Access publishing; we are looking forward to publishing your work in PLOS Global Public Health.

Best regards,

Andrés F. Henao-Martínez, M.D.

Academic Editor

PLOS Global Public Health

***********************************************************

Reviewer Comments (if any, and for reference):

Reviewer's Responses to Questions

**Comments to the Author**

Reviewer #1: All comments have been addressed

Reviewer #2: All comments have been addressed

Reviewer #3: All comments have been addressed

**********

publication criteria?

Reviewer #1: Yes

Reviewer #2: Yes

Reviewer #3: Yes

**********

3. Has the statistical analysis been performed appropriately and rigorously?

Reviewer #1: Yes

Reviewer #2: Yes

Reviewer #3: Yes

**********

4. Have the authors made all data underlying the findings in their manuscript fully available (please refer to the Data Availability Statement at the start of the manuscript PDF file)?

The PLOS Data policy

Reviewer #1: Yes

Reviewer #2: Yes

Reviewer #3: Yes

**********

5. Is the manuscript presented in an intelligible fashion and written in standard English?

Reviewer #1: Yes

Reviewer #2: Yes

Reviewer #3: Yes

**********

Reviewer #1: The authors have made several changes, and I am satisfied. However, I do think table 2,3 and 4 are not necessary. The text in the results is enough.

All my questions have been answered.

Reviewer #2: Thank you for addressing all the reviewers comments and doing corrections/revisions to the sections the reviewers had concerns about. You have addressed all of my concerns and I do not have any other recommendations.

Reviewer #3: Thank you for your work and revision. Now is much better. Good luck.

**********

what does this mean?). If published, this will include your full peer review and any attached files.

**Do you want your identity to be public for this peer review?**  If you choose “no”, your identity will remain anonymous but your review may still be made public.

For information about this choice, including consent withdrawal, please see our Privacy Policy

Reviewer #1: **Yes:**  Siv Karlsson Stafseth

Reviewer #2: **Yes:**  Elizabeth Scruth

Reviewer #3: **Yes:**  Olga Cerela

**********
